# Flexible and Wearable Sensors—Design, Fabrication Methods, and Applications

**DOI:** 10.3390/s26072196

**Published:** 2026-04-02

**Authors:** Yichun Ding, Tingting Zhao, Ziran Wang

**Affiliations:** 1 School of Physics and Materials Science, Nanchang University, Nanchang 330031, China; 2 Key Laboratory of Advanced Display and System Applications, Ministry of Education, School of Mechatronic Engineering and Automation, Shanghai University, Shanghai 200444, China; ttzhao@shu.edu.cn; 3 Key Laboratory of High Efficiency and Clean Mechanical Manufacture, Ministry of Education, School of Mechanical Engineering, Shandong University, Jinan 250000, China; wangziran@sdu.edu.cn

## 1. Introduction

The rapid advancement of the Internet of Things (IoT), artificial intelligence (AI), and personalized healthcare has catalyzed an unprecedented demand for flexible and wearable sensor technologies [1,2]. Unlike conventional rigid sensors, flexible and wearable sensors offer unique advantages, including lightweight construction, excellent mechanical compliance, high stretchability, and the ability to conform to irregular surfaces such as human skin [3,4]. These distinctive characteristics have positioned flexible sensors as key enablers for a wide range of emerging applications, including electronic skin, human–machine interfaces, continuous health monitoring, sports performance tracking, and soft robotics [5,6,7,8]. The convergence of innovative materials science, advanced fabrication techniques, and intelligent system integration has accelerated the development of next-generation wearable sensors that are not only high-performing but also comfortable, durable, and scalable for real-world deployment [9,10,11,12]. This Special Issue, “Flexible and Wearable Sensors: Design, Fabrication Methods, and Applications”, presents a collection of cutting-edge research and comprehensive review articles that address critical challenges and highlight recent breakthroughs in the field, spanning from fundamental material design to practical application demonstrations.

## 2. An Overview of Published Articles

A total of eighteen manuscripts were submitted for consideration for this Special Issue, all of which underwent a rigorous review process. Ultimately, eleven papers were accepted for publication and inclusion, comprising seven research articles and four reviews, each offering unique insights into different aspects of flexible and wearable sensor technology.

As summarized in Table 1, the contributions cover a wide spectrum of flexible and wearable sensor technologies, encompassing diverse material systems, fabrication methods, and application domains. Contributions 1, 2, 3, 5, 6, 7, 9, and 11 focus on the development of high-performance flexible sensors utilizing various transduction mechanisms, including piezoresistive strain sensors based on silver nanowire–polyurethane composites, iontronic pressure sensors with engineered fiber microstructures, Kirigami-structured piezoelectric sensors for heart sound monitoring, capacitive electrodes for non-contact ECG, and triboelectric nanogenerators (TENGs) for mechanical energy harvesting and self-powered sensing. In detail, contributions 4 and 8 explore the integration of wearable sensors into novel application areas such as musical expression and provide comprehensive reviews of design and fabrication methodologies; contributions 6 and 7 emphasize advanced computational approaches, including knowledge graph-based sensor design and time-reversal algorithms for ultrasound focusing; contribution 9 specifically reviews electrospun thermoplastic polyurethane (TPU) strain sensors and their applications; and contribution 10 provides a focused review on optical fiber sensors for rehabilitation monitoring. Below are specific highlights for each contribution.

Shan et al. (contribution 1) present a systematic study on the preparation and applications of silver nanowire and TPU nanofibers for flexible strain sensors. By synthesizing ultra-high-aspect-ratio silver nanowires (aspect ratio > 1500) and combining them with electrospun TPU nanofiber membranes in a sandwich structure, the authors achieved a flexible sensor with exceptional properties: low initial resistance (26.7 Ω), high sensitivity (gauge factor > 19.21), rapid response time (112 ms), and remarkable cycling stability over 3000 stretching cycles. The sensor demonstrated a stable electrical signal output for applications in motion monitoring, human–computer interaction, and healthcare.

Liu et al. (contribution 2) conduct a comparative investigation into the impact of microstructure on the sensing performance of fiber-based iontronic pressure sensors. Using four distinct fiber materials (woven fabric, cotton nonwoven, viscose spunlace, and electrospun TPU nanofiber mats), the authors systematically evaluated how fiber diameter, porosity, and thickness influence ionic liquid absorption and consequent sensing performance. The optimized sensor, fabricated with a 33 μm-thick TPU/IL nanofiber mat, achieved remarkable sensitivities of 3.10 kPa^−1^, 1.85 kPa^−1^, and 1.02 kPa^−1^ across pressure ranges of 0–200 kPa, 200–400 kPa, and 400–700 kPa, respectively, with an ultrafast response time of 2.71 ms. This work provides valuable guidance for selecting and utilizing fiber materials to develop high-sensitivity iontronic pressure sensors for wearable electronics.

Zhang et al. (contribution 3) introduce a stretchable piezoelectric sensor with a Kirigami-inspired design for heart sound monitoring. The five-layer sandwich structure (PI–electrode–PVDF–electrode–PI) incorporates periodic Kirigami cuts that significantly enhance stretchability while maintaining piezoelectric conversion efficiency. Finite element simulations revealed that the Kirigami structure’s stretchability is more sensitive to hinge length and thickness than to hinge width. The sensor exhibited excellent stability, with a maximum amplitude variation of approximately 11% under 0–30% strain and less than 9% output fluctuation during 1200 s of continuous excitation. Seven-day monitoring confirmed reliable detection of the first (S1) and second (S2) heart sounds, demonstrating its potential for wearable long-term cardiovascular monitoring.

Wexler et al. (contribution 4) explore the innovative concept of wearable technologies as a medium for musical expression. The authors developed a wearable device equipped with 32 touch-sensitive pressure sensors, a nine-axis inertial measurement unit motion sensor, and LED and vibrational haptic feedback components, which can be worn as a wrist strap, hand strap, or surface drum pad. The device translates hand motions and gestures into musical outputs, offering an intuitive and accessible interface for music creation. Through iterative prototyping and user evaluation, the study demonstrates how wearable technology can democratize music learning and expression, bridging the accessibility gap in musical education and performance.

Iliev et al. (contribution 5) present the design and assessment of flexible capacitive electrodes for reusable ECG monitoring, with particular attention to the effects of sweat and adapted front-end configuration. Using a simple, cost-effective multilayer structure consisting of a copper conductor laminated with a polyimide dielectric layer on a polyurethane support, the authors evaluated electrode performance under varying textile moisture levels and exposure to common disinfectants. Electrochemical impedance spectroscopy confirmed the stable capacitive behavior and chemical stability of the polyimide–copper interface. A custom front-end readout circuit incorporating digitally adjustable gain and baseline correction enabled robust through-textile ECG signal acquisition, demonstrating the feasibility of this low-cost, reusable electrode platform for wearable biosignal monitoring.

Ke et al. (contribution 6) propose a data-driven approach to strain sensor design using a knowledge graph framework. By constructing a knowledge graph that captures relationships among materials, structures, fabrication methods, and performance metrics from the published literature, the authors employed graph representation learning to predict sensor properties and discover new design combinations. The framework achieved a prediction precision up to 0.81 and successfully identified a TPU/graphene/carbon black combination with a high correlation (0.93). The designed sensor demonstrated a wide strain range (300%) and closely matched the predicted performance, showcasing the potential of AI-assisted approaches to accelerate sensor development and reduce trial-and-error experimentation.

Jia et al. (contribution 7) report a flexible ultrasound transducer with a controllable acoustic field enabled by a unique time-reversal algorithm. The device incorporates a low-impedance piezoelectric material and an island-bridge structure, with each pixel achieving a consistent sound pressure of approximately 36 kPa. To address the challenge of accurate focusing when the device is bent, the authors developed a Virtual Point Source Time-Reversal Algorithm (VPSTRA) that compensates for time differences in ultrasound transmission through the skull. The simulation results demonstrated successful focal formation at both single and multiple virtual source points, offering a promising approach for non-invasive brain disease treatment and deep-tissue sensing.

Ali et al. (contribution 8) provide a thorough overview of wearable and flexible sensor devices, focusing on recent advances in designs, fabrication methods, and applications. The review covers state-of-the-art wireless communication technologies for wearable devices, smart wearable sensors for physiological monitoring, and emerging additive manufacturing techniques including direct ink writing (DIW) and 3D to 6D printing. The authors also discuss the importance of human body phantoms for evaluating wearable device performance and address challenges related to biocompatibility, power supply, and specific absorption rate (SAR) considerations for next-generation healthcare applications.

Zhou et al. (contribution 9) present a focused review on flexible strain sensors based on TPU nanofibers fabricated using electrospinning. The authors systematically summarize recent advances in incorporating various conductive fillers—including carbonaceous materials (carbon black, carbon nanotubes, and graphene), MXene, metallic nanomaterials (silver nanowires, liquid metals), and conductive polymers (PPy, PEDOT:PSS, and PANI)—into electrospun TPU nanofibers. The review discusses the working principles of strain sensors and electrospinning technology, highlights strategies for optimizing sensor performance, and explores applications in motion detection, health monitoring, human–computer interaction, and electronic skin.

Li et al. (contribution 10) review wearable optical fiber sensors for rehabilitation monitoring. The authors introduce the principles and technologies of various fiber sensors, including Fiber Bragg Grating (FBG) sensors, self-luminescent stretchable optical fiber sensors, Fabry–Perot sensors, and polymer optical fiber sensors. The review discusses specific applications in detecting finger movements, physiological parameters (blood oxygen saturation, pulse waveform, and skin temperature), and tactile responses during rehabilitation. The authors also address current challenges and future research directions, including improving sensitivity and flexibility, reducing costs, and enhancing system integration for clinical applications.

Punnarao and Yoon (contribution 11) present a review of flexible triboelectric mechanical energy harvesters for wearable and self-powered sensing applications. The review explains the working principles and four operational modes of TENGs (contact–separation, single-electrode, lateral-sliding, and freestanding modes), with detailed simulation results and working mechanisms. The authors systematically analyze fabrication techniques for triboelectric flexible films, including hydrothermal synthesis, electrospinning, 3D printing, and spray coating. The review covers applications in mechanical energy harvesting, self-powered sensors for human motion detection, healthcare monitoring, and human–machine interaction, while also addressing the current limitations and future directions for the field.

## 3. Conclusions

The collection of articles in this Special Issue reflects the remarkable progress and interdisciplinary nature of flexible and wearable sensor research. From the development of high-performance materials, such as silver nanowires and nanofibers, to innovative structural designs, including Kirigami patterns and sandwich architectures, these contributions demonstrate the diverse strategies being pursued to enhance sensor performance, flexibility, and wearability. Advances in fabrication methods, ranging from electrospinning and direct ink writing to 3D and 4D printing, are enabling the scalable production of sensors with precisely controlled microstructures and multifunctional capabilities. The integration of these sensors into practical applications, including cardiac monitoring, rehabilitation, musical expression, and human–machine interaction, illustrates their transformative potential across healthcare, entertainment, and beyond. Moreover, the emergence of data-driven approaches, such as knowledge graph-based design and machine learning-assisted signal processing, promises to accelerate innovation and enable the rational design of next-generation wearable sensors. While challenges remain, including long-term stability, biocompatibility, power supply, and cost-effective manufacturing, the collective efforts of researchers worldwide are steadily addressing these obstacles. We anticipate that continued advancements in materials science, manufacturing technologies, and system integration will further expand the capabilities and adoption of flexible and wearable sensors, ultimately improving quality of life through personalized, continuous, and unobtrusive monitoring and interaction.

## Figures and Tables

**Table 1:** Analysis of the published contributions in the Special Issue.

# of Contribution	Research Area (Type of Sensor)	Focus	Type of Article
1	Piezoresistive strain sensor	Materials (conductive silver nanowires, electrospun nanofibers as substrate); human motion monitoring	Research
2	Iontronic pressure sensor	Materials (iontronic fiber materials); human motion monitoring	Research
3	Piezoelectric pressure sensor	Structure design (enabling stretchability by Kirigami design); finite element simulations; heart sound monitoring	Research
4	Piezoresistive strain sensor, IMU motion sensor	Musical expression (translates hand motions and gestures into musical outputs)	Research
5	Capacitive electrode for ECG acquisition	Materials (multilayer Kapton-insulated Cu foil structure on a flexible polyurethane structural support); ECG signal acquisition	Research
6	Piezoresistive strain sensor	Data-driven (machine learning) materials exploration for strain sensor; performance prediction; human motion monitoring	Research
7	Flexible ultrasound transducer	Structure design and device fabrication (piezoelectric material and island-bridge device structure); time-reversal algorithm; transcranial focused ultrasound simulation	Research
8	Versatile sensors; integrated sensors	Fabrication methods; human body phantoms for evaluating wearable device performance	Review
9	Piezoresistive strain sensor	Conductive materials; electrospun; polyurethane; motion detection; healthcare monitoring; human–machine interaction	Review
10	Optical fiber sensors	Working principle of wearable optical fiber sensor; Fiber Bragg Grating sensors, self-luminescent stretchable optical fiber sensors, Fabry–Perot sensors, and polymer optical fiber sensors	Review
11	Triboelectric nanogenerators (TENGs)	Working principles and operational modes of TENGs; applications (mechanical energy harvesting, self-powered sensors for human motion detection, healthcare monitoring, and human–machine interaction)	Review
